# Integrative analysis of gene expression profiles of substantia nigra identifies potential diagnosis biomarkers in Parkinson's disease

**DOI:** 10.1038/s41598-024-52276-0

**Published:** 2024-01-25

**Authors:** Junming Huang, Bowen Li, Huangwei Wei, Chengxin Li, Chao Liu, Hua Mi, Shaohua Chen

**Affiliations:** 1 Department of Urology, Guangxi Medical University Cancer Hospital, Nanning, 530000 Guangxi China; 2 Department of Urology, The First Affiliated Hospital of Guangxi Medical University, Nanning, 530000 Guangxi China; 3 Department of Neurology, The People Hospital of Guangxi Zhuang Autonomous Region, Nanning, Guangxi China; 4 Department of Neurology, The First Affiliated Hospital of Guangxi Medical University, Nanning, Guangxi China

**Keywords:** Genetics, Immunology, Neuroscience

## Abstract

Parkinson's disease (PD) is a progressive neurodegenerative disease whose etiology is attributed to development of Lewy bodies and degeneration of dopaminergic neurons in the substantia nigra (SN). Currently, there are no definitive diagnostic indicators for PD. In this study, we aimed to identify potential diagnostic biomarkers for PD and analyzed the impact of immune cell infiltrations on disease pathogenesis. The PD expression profile data for human SN tissue, GSE7621, GSE20141, GSE20159, GSE20163 and GSE20164 were downloaded from the Gene Expression Omnibus (GEO) database for use in the training model. After normalization and merging, we identified differentially expressed genes (DEGs) using the Robust rank aggregation (RRA) analysis. Simultaneously, DEGs after batch correction were identified. Gene interactions were determined through venn Diagram analysis. Functional analyses and protein–protein interaction (PPI) networks were used to the identify hub genes, which were visualized through Cytoscape. A Lasso Cox regression model was employed to identify the potential diagnostic genes. The GSE20292 dataset was used for validation. The proportion of infiltrating immune cells in the samples were determined via the CIBERSORT method. Sixty-two DEGs were screened in this study. They were found to be enriched in nerve conduction, dopamine (DA) metabolism, and DA biosynthesis Gene Ontology (GO) terms. The PPI network and Lasso Cox regression analysis revealed seven potential diagnostic genes, namely *SLC18A2, TAC1, PCDH8, KIAA0319, PDE6H, AXIN1, and AGTR1,* were subsequently validated in peripheral blood samples obtained from healthy control (HC) and PD patients, as well as in the GSE20292 dataset. The results revealed the exceptional sensitivity and specificity of these genes in PD diagnosis and monitoring. Moreover, PD patients exhibited a higher number of plasma cells, compared to HC individuals. The *SLC18A2, TAC1, PCDH8, KIAA0319, PDE6H, AXIN1*, and *AGTR1* are potential diagnostic biomarkers for PD. Our findings also reveal the essential roles of immune cell infiltration in both disease onset and trajectory.

## Introduction

Over the last 25 years, there has been a global increase in the number of individuals affected by, dying from, or suffering from long-term neurological disorder-associated disabilities. This is despite the development of multiple novel diagnostic and treatment methods^1^. Parkinson’s disease (PD) is a significant contributor to neurological disability, and is the second most prevalent neurodegenerative disorder worldwide. With a global prevalence exceeding 6 million individuals, the condition has exhibited a remarkable 2.5-fold rise in occurrence over the past generation^2^. In industrialized countries, the estimated prevalence of this condition is 0.3%, which is rarely observed in patients under the age of 40, but its incidence is increasing with advancing age^3^. The disease is associated with typical motor symptoms, such as parkinsonism, Lewy bodies and dopaminergic neuron degeneration in the substantia nigra (SN)^4–6^. The underlying etiologic factors for this complex disease are attributed to a combination of genetic and environmental factors that affect essential cellular processes^7,8^. Clinical management of this disease is challenged by limitations of treatment and definitive diagnosis, particularly at the earliest stages of the disease.

Through transcriptomic analysis, studies have identified various differentially expressed genes (DEGs) and dysregulated pathways. Beta-glucocerebrosidase is involved in both the endo-lysosomal pathway and immune responses, which are two critical processes in PD development^9^. Kurvits et al. used the Robust rank aggregation (RRA) strategy and found that *IL18R1,* an interleukin receptor associated with proinflammatory responses, was induced by GM-CSF administration and was associated with neuroprotective mechanisms in PD ^10,11^. Therefore, PD is a potential multisystem disorder. Songyun Zhao et al^12^. Performed Lasso Cox regression analysis and constructed a model that yielded the most substantial net gain, underscoring the critical role of the advanced risk model in guiding personalized anti-cancer therapy and driving informed decision-making, which is related to PD. Protein–protein interaction (PPI) network analysis is a powerful approach for achieving a comprehensive understanding of biological processes at molecular and systemic levels^13^. For downstream applications, the STRING database integrates data from various primary databases^14^. Recently, Kim et al. reported on the functional significance of thiol-oxidoreductase TXNIP in development of LRRK2-associated PD within a three dimensional (3D) environment, highlighting the potential of 3D organoid-based models in advancing therapeutic discovery for sporadic PD^15^. The significance of immune cell infiltrations in PD onset and progression has been well-established^16,17^. CIBERSORT, a computational tool, allows for assessment of immune cell proportions based on gene expression data^18^. Both innate and adaptive immune systems play a role in neuronal death and PD pathogenesis^19^. Despite remarkable advances in recent years, the etiopathogenesis of PD, encompassing the contribution of biomarkers and underlying biological processes leading to formation of Lewy bodies and dopaminergic neuron loss in the SN, has not been fully elucidated. In this study, we aimed at identifying the potential biomarkers and molecular mechanisms that can promote healthy brain functions and avert PD onset.

We downloaded five datasets from the Gene Expression Omnibus (GEO), combined them as the training set and used another dataset as the validation set. Then, RRA and batch correction were used in the training set to establish the consensus DEGs of PD. Next, Gene Ontology (GO) and the Kyoto Encyclopedia of Genes and Genomes (KEGG) pathway enrichment analyses were performed to reveal the biological functions of the DEGs. The PPI networks were used to establish interactions among DEGs-related proteins. Lasso Cox regression models were used to identify the possible biomarkers for PD. Finally, 7 genes, namely *SLC18A2*, *TAC1*, *PCDH8*, *KIAA0319*, *PDE6H*, *AXIN1*, and *AGTR1,* were identified to be potential diagnostic genes for PD. To validate the clinical accuracy of the genes, a validation set and reverse transcription-quantitative real-time polymerase chain reaction (RT-qPCR) were performed. CIBERSORT analysis was performed to investigate immune cell infiltrations in PD samples. The goal of this study was to identify the potential diagnostic genes for PD and to characterize immune cell infiltrations in peripheral blood samples from PD patients.

## Materials and methods

### Data source and pre-processing.

**Figure 1:**
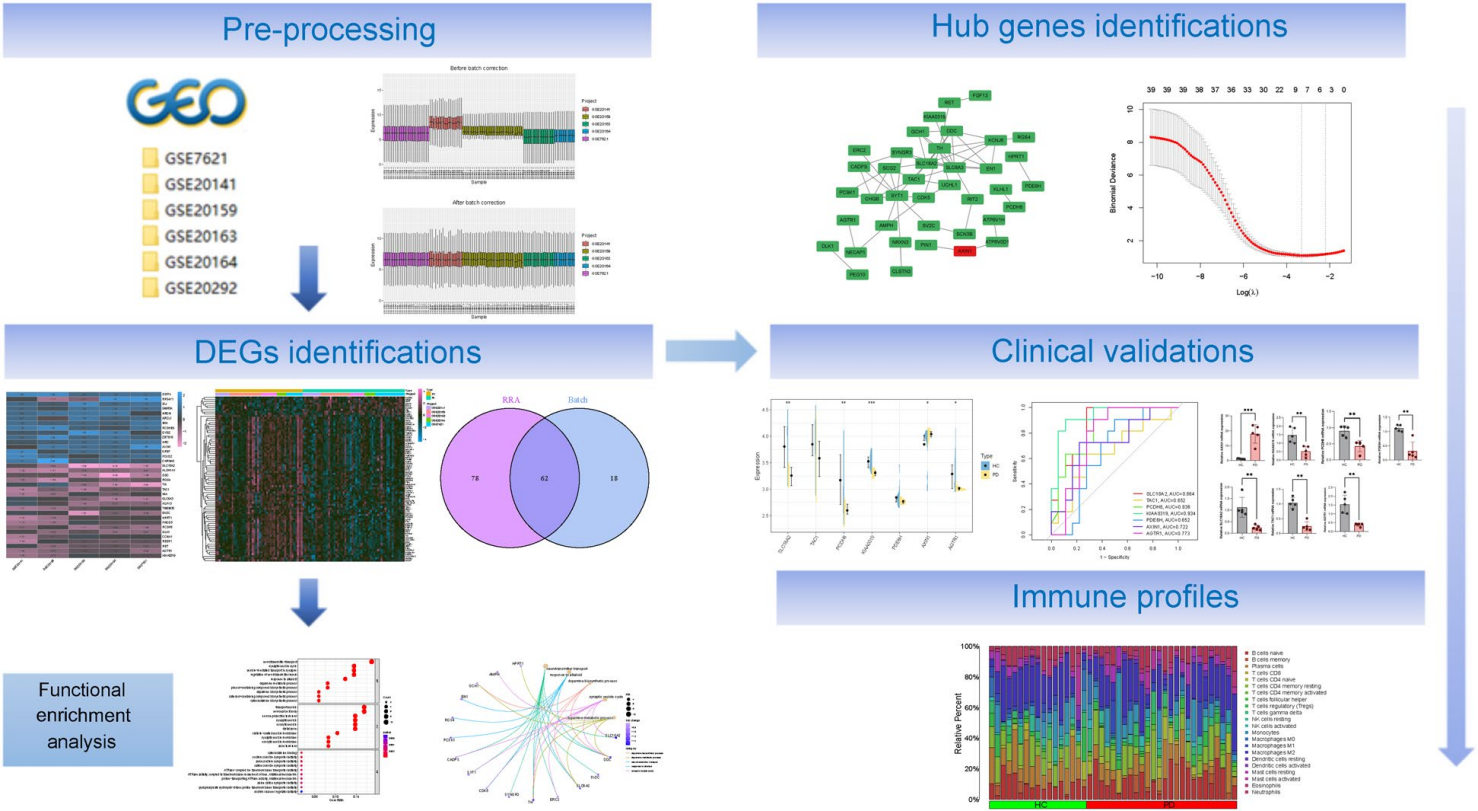
Flowchart for bioinformatics analysis in this study.

Five PD datasets (GSE7621, GSE20141, GSE20159, GSE20163 and GSE20164) were downloaded from the GEO database (https://www.ncbi.nlm.nih.gov/geo/), merged and used as the training set, which contains 48 healthy control (HC) and 56 PD patients. The GSE20292 dataset, which consists of transcriptional analysis data for the whole SN in PD, was used for validation (Table 1). The ‘sva’ package (https://bioconductor.org/packages/release/bioc/html/sva.html) was used to remove batch effects from the training sets. The principal component analysis (PCA) cluster plot was used to visualize the effects of removing inter-batch differences. The workflow of this study is shown in Fig. 1.

**Table 1:** Dataset characteristics.

Dataset	Platform	Type	No. of samples	Sample source	Age	Gender female: male	Country
GSE7621	GPL570	Microarrays	25 (9 HCs, 16 PDs)	Substantia nigra	–	8:17	United States
GSE20141	GPL570	Expression profiling by array	18 (8 HCs, 10 PDs)	Dopaminergic neuron and substantia nigra transcriptomes	–	–	United States
GSE20159	GPL6947	Expression profiling by array	33 (17 HCs, 16 PDs)	Snap-frozen human substantia nigra	HC (40-95y); PD (56-103y)	16:17	Britain
GSE20163	GPL96	Expression profiling by array	17 (9 HCs, 8 PDs)	Substantia nigra	HC (52-84y); PD (70-84y)	–	Britain
GSE20164	GPL96	Expression profiling by array	11 (5 HCs, 6 PDs)	Substantia nigra	HC (72-90y); PD (74-89y)	6:5	Britain
GSE20292	GPL96	Expression profiling by array	29 (18 HCs, 11 PDs)	substantia nigra	HC (41-94y); PD (67-84y)	10:19	United States

RRA analysis.

The RRA analysis was employed to systematically integrate gene expression information across diverse datasets^20^. We integrated the R packages ‘limma’ (https://bioconductor.org/packages/release/bioc/html/limma.html) and ‘RobustRankAggreg’ (https://cran.r-project.org/web/packages/RobustRankAggreg/index.html) to identify genes that exhibited statistically significant changes (adjusted *P* value < 0.05) and those that were statistically significance in adjusted *P* value < 0.05 and |log fold change (FC) |> 1.0.

### Functional enrichment analysis.

The GO and KEGG enrichment analyses of DEGs were conducted using the ‘clusterProfiler’ (https://bioconductor.org/packages/release/bioc/html/clusterProfiler.html) package of R software. Findings of GO annotation analysis were categorized as follows: Biological Process (BP), Molecular Function (MF), and Cellular Components (CC)^21,22^. Significant enrichment was defined as adjusted *P* values < 0.05.

### Establishment of the PPI network and identification of hub genes.

To establish the interplay of DEGs, PPI network analysis was performed using the STRING database (https://cn.string-db.org/). A stringent interaction score threshold of 0.4 was employed to identify the most reliable and relevant interactions among the DEGs. Genes were denoted by nodes while connections between them were represented by edges. Subsequently, the main regulatory network was constructed and visualized using Cytoscape (https://cytoscape.org/, version 3.9.1). The cytoHubba plugin of Cytoscape was used to identify the hub genes in the PPI network. A systematic evaluation of central genes was performed using a comprehensive repertoire of ten distinct methods: MCC, DMNC, Degree, BottleNeck, EcCentricity, Closeness, MNC, Radiality, Stress, andBetweenness. By synthesizing the results obtained from these diverse approaches, the top 39 hub genes were identified, representing a collective selection based on their high-ranking positions across the ten methods.

### Identification of PD biomarkers via Lasso Cox regression analysis.

The Lasso Cox regression analysis, a penalized regression technique, is effective for predicting outcomes and has low correlations, making it suitable for selecting the most relevant features in datasets with various variables. The ‘glmnet’ package(https://cran.r-project.org/web/packages/glmnet/) was used to extract and fit the consensus DEGs expression profiles into the Lasso Cox regression model. To identify the DEGs between HC and PD patients after Lasso Cox regression analysis, a heatmap and volcano map were drawn in the training set.

### Evaluation of the diagnostic model in verification set.

A heatmap and a violin map were drawn to identify the differentially expressed hub genes between HC and PD patients in the verification set. Then, the efficacy of distinguishing between HC and PD patients was tested by receiver operating characteristic (ROC) curve analysis.

### Immune cell infiltration analysis.

The R package ‘CIBERSORT’ (https://github.com/Moonerss/CIBERSORT) was used to calculate the fractions of immune infiltrating cells in the training set, which were visualized using the boxplot. The Violin Plot was used to identify statistically significant differences in immune cells between HC and PD patients (*P* value < 0.05).

### RT-qPCR

Independent peripheral blood samples from PD patients were used to assess the practical efficacy of our diagnostic model. Clinical whole blood samples were collected from both HC and PD patients, and total DNA were extracted using the FastPure Blood DNA Isolation Mini Kit V2. The β-Actin primer pair was used as the internal control. Relative gene expressions were calculated and normalized via the Ct technique and ΔΔCt method. Clinical information for PD patients used in the RT-qPCR is detailed in Supplementary Table S1. Primer sequences are detailed in Table 2.

**Table 2:** Primer sequences used in RT-qPCR.

Gene	Primer direction	Sequence
SLC18A2	Forward	TGCTCACTGTCGTGGTCCC
	Reverse	TGTGCTGTGTGGCGGTCT
TAC1	Forward	ACCAGAGAAACTCAGCACCCC
	Reverse	ACAAGAAAAAAGACTGCCAAGG
PCDH8	Forward	CCTCTGCTGGGTGCTCTCA
	Reverse	ACTCTCGTGGGTCGTCTCC
KIAA0319	Forward	CTCACACCTTCCCTGTCGTAGA
	Reverse	GAGCCCCTGTTCAGCATCA
PDE6H	Forward	ACAACACTACTCTGCCTGCTCC
	Reverse	CATCTCCAAATCCTTTCACACC
AXIN1	Forward	GACCTGGGGTATGAGCCTGA
	Reverse	GGCTTATCCCATCTTGGTCATC
AGTR1	Forward	ATTGCCTGAATCCTCTTTTTTATG
	Reverse	ATTATCTGAGGGGCGGTAGG

### Statistical analysis

Data preparation, functional analysis, and modeling were performed using R software (https://cran.r-project.org/, v4.1.3). All *P* values were two-sided and differences with *P* < 0.05 and log |FC|< 0.585 were considered significant. Cytoscape was utilized to visualize the PPI network. ROC curves analysis was derived using ‘pROC’ packages (https://cran.r-project.org/web/packages/pROC/). Intergroup comparisons were performed via the Wilcox test.

### Ethical approval

In this study, we confirm that all experiments and methods were conducted strictly in accordance with relevant guidelines and regulations. The experimental protocols have been approved by the ethics committee to ensure the legality and ethical compliance of the research. Hereby declare that informed consent was obtained from all human participants, including the use of tissue samples, involved in our research study. The human tissue sample collection for this study have obtained approval from the following institution: The first affiliated hospital of guangxi medical university. The number of the approving: 2023-E258-01.

## Results

### Identification of DEGs.

**Figure 2:**
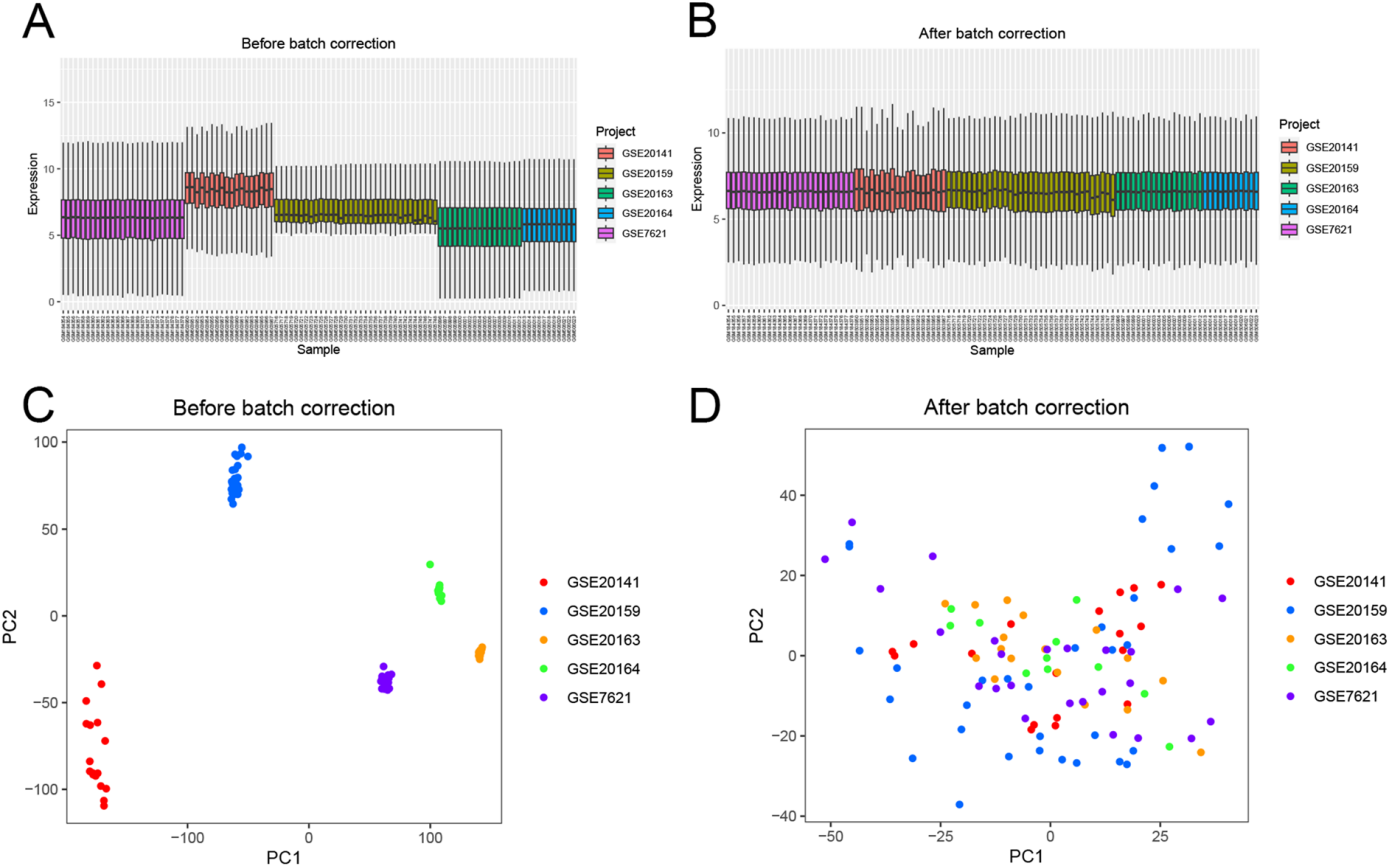
The datasets were normalized, and the batch effects removed. (**A**,**B**) Boxplot of batch effects of combined sets before and after normalization. (**C**, **D**) A two-dimensional PCA cluster plot of datasets before and after batch effects removal.

**Figure 3:**
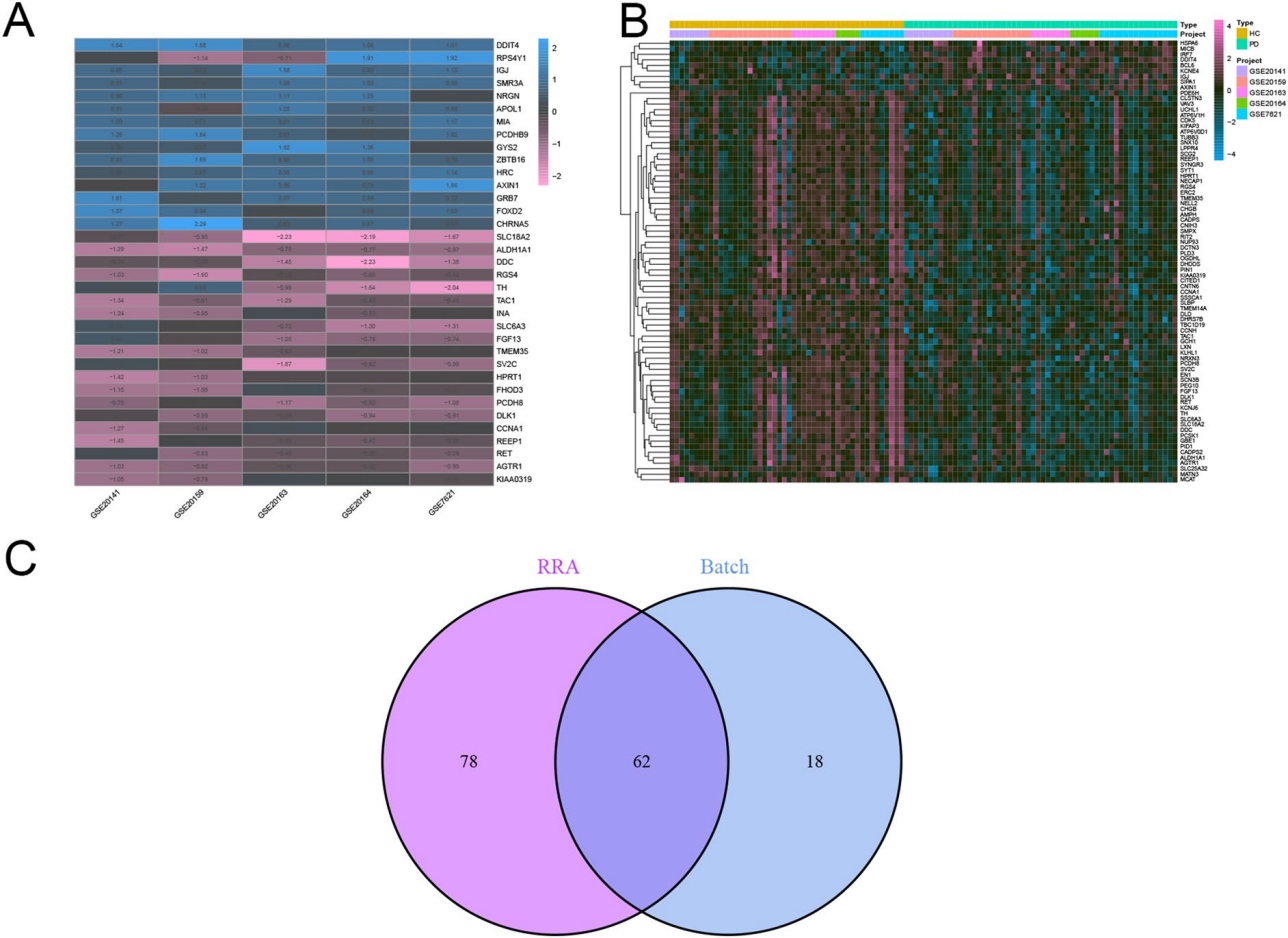
Identification of DEGs. (**A**) RRA analysis identified the DEGs. P value-based heatmap showing the top 35 upregulated and downregulated genes. (**B**) Heatmap of DEGs in HC and PD patients. (**C**) Venn diagram of DEGs between RRA analysis and batch correction.

Five datasets (GSE7621, GSE20141, GSE20159, GSE20163 and GSE20164) with a total of 104 samples were included as the training set. Batch correction based on the ‘sva’ R package was performed to diminish the potential for bias to be introduced by batch effects on subsequent analyses. Boxplot and PCA revealed the differences before and after batch correction, indicating the successful removal of batch effects (Fig. 2A–D). The R package ‘RobustRankAggreg’ was used to perform the RRA analysis (Fig. 3A). Batch correction was performed on the training set (Fig. 3B). A total of 80 DEGs (nine upregulated and 71 downregulated) between HC and PD patients were identified via RRA and batch correction. Based on the R package of ‘venn’ (https://cran.r-project.org/web/packages/venn/), There are 62 DEGs were detected between RRA analysis and batch correction. (Fig. 3C). In conclusion, by integrating the datasets and treating them as the training set, we were able to filter out 62 DEGs using RRA and batch correction, thereby laying the foundation for subsequent analyses.

### GO and KEGG pathway functional enrichment analysis of DEGs.

**Figure 4:**
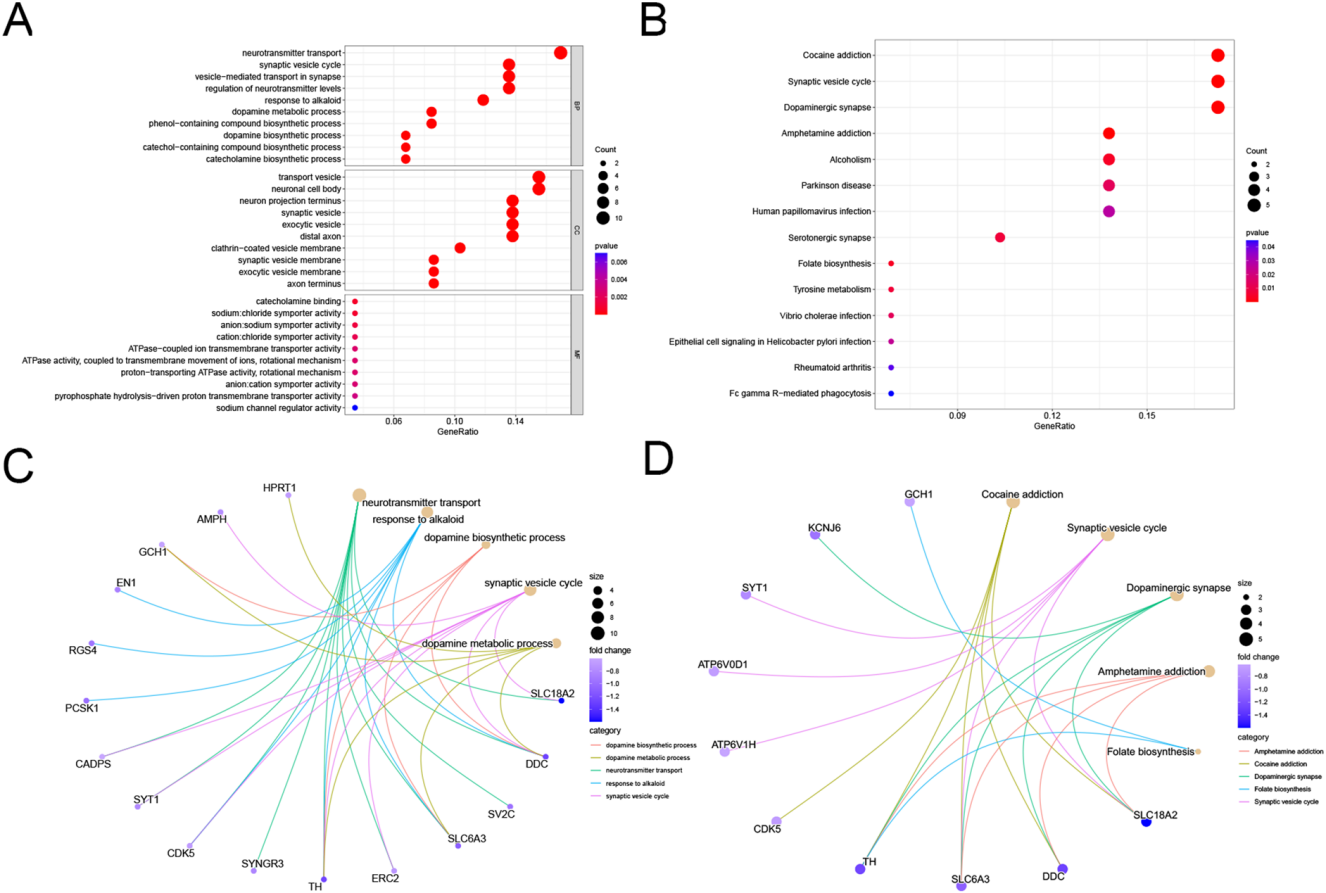
The GO analysis and KEGG pathway analysis of DEGs. (**A**) Dot plot showing GO analysis of DEGs. (**B**) Dot plot showing KEGG pathway analysis of DEGs. (**C**) Circle graph showing the DEGs that were enriched in the top 5 GO categories of BPs. (**D**) Circle graph showing the DEGs that were enriched in KEGG pathway analysis.

The GO and KEGG enrichment analyses were performed to determine the functions of the 62 significant DEGs (Fig. 4A,B). The enriched GO terms were nerve conduction, dopamine (DA) metabolism, and DA biosynthesis. The enriched KEGG terms included PD and the cycle of nerve conduction. Association of proteins and the top five GO and KEGG terms were visualized using a cnetplot (Fig. 4C,D). Based on our analysis, it is possible that the identified DEGs are closely associated with the nervous system and may potentially impact PD pathogenesis.

### PPI network analysis and hub genes selection

**Figure 5:**
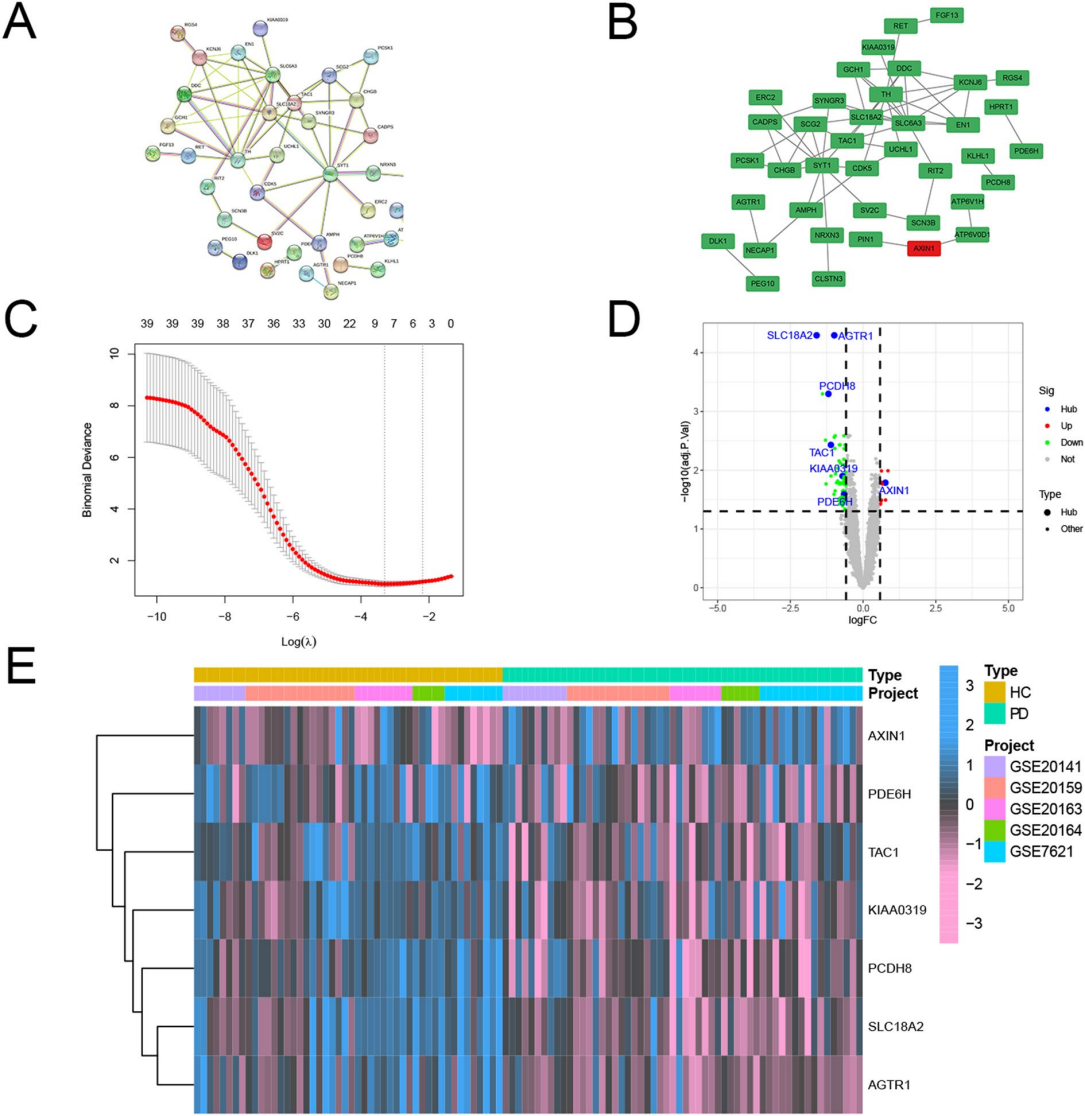
PPI network and hub gene selection. (**A**) The top 62 hub genes in the PPI network of DEGs based on node degree. (**B**) Hub genes were identified by taking the interplay of the first 39 genes in the ten classification methods of cytoHubba. (**C**) Hub gene selection in the Lasso Cox regression model. Vertical lines were drawn at optimal values by the minimum criteria and the 1–SE criteria. (**D**) The hub gene landscape in the volcano map. © The heatmap of hub genes in HC and PD patients.

The PPI networks were analyzed in the STRING online database to elucidate on protein interactions among the DEGs (Fig. 5A). Subsequently, the main regulatory network was constructed and visualized using Cytoscape Consequently, a total of 39 genes were identified (Fig. 5B). Ranked and networks of the genes for each approach were listed in Supplementary Table S1 and Supplementary Figure S1. Expression profiles of the hub genes were extracted and integrated into the Lasso Cox regression model to identify the possible biomarkers for PD. To build a model that facilitates quantification of each patient with accuracy, seven of the 39 DEGs, including *SLC18A2*, *TAC1*, *PCDH8*, *KIAA0319*, *PDE6H*, *AXIN1*, and *AGTR1* were retained by application of the Lasso Cox regression model with a minimum of λ (Fig. 5C). The distribution of these genes is presented in the volcano plot (Fig. 5D). Expression levels of the above genes significantly differed between the two sets as shown in the heatmap (Fig. 5E). These findings imply that the hub genes may have pivotal functions in biological mechanisms underlying PD and are promising therapeutic targets.

### Immune cell infiltration analysis.

**Figure 6:**
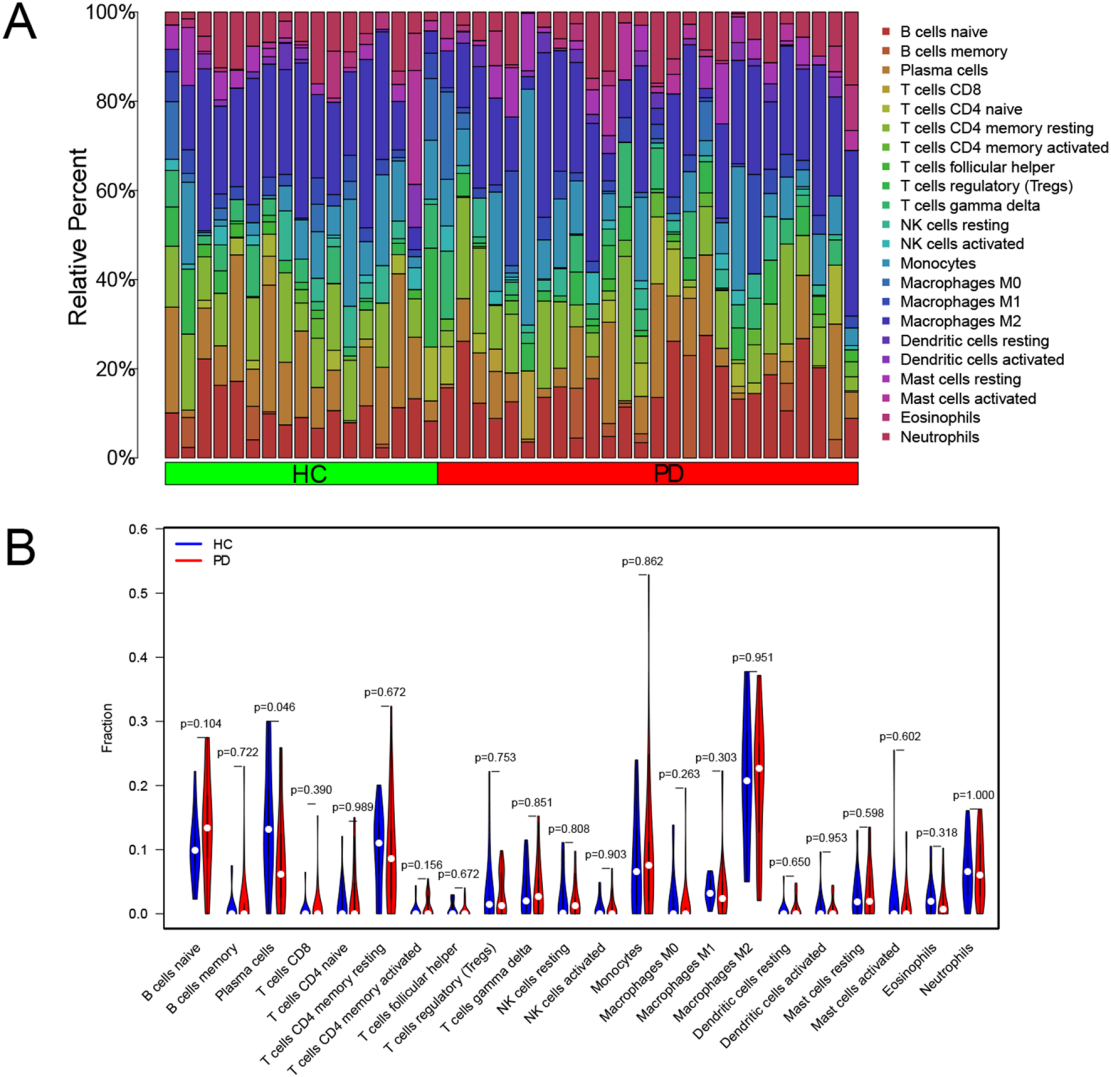
CIBERSORT tumor-infiltrating immune cell analysis. (**A**) The proportion of 22 immune cells infiltrating the samples. (**B**) A violin plot of differential abundance of infiltrating immune cells between HC and PD patients.

Progression of neurodegenerative processes in PD may be sustained by changes in immune cell markers that induce or worsen neuroinflammation^6,16^. Therefore, to establish the constitution of immune cells in PD samples for the purpose of obtaining the difference between HC and PD patients in relative abundance of immune cells, the immune status was evaluated. Constituent ratios for 22 infiltrating immune cell types were as shown in Fig. 6A. The PD patients exhibited higher counts of plasma cells compared with HC (Fig. 6B). The intensity of plasma cell expressions, as evidenced by the image, was closely associated with PD.

### Performance of the diagnostic model in SN datasets and samples.

**Figure 7:**
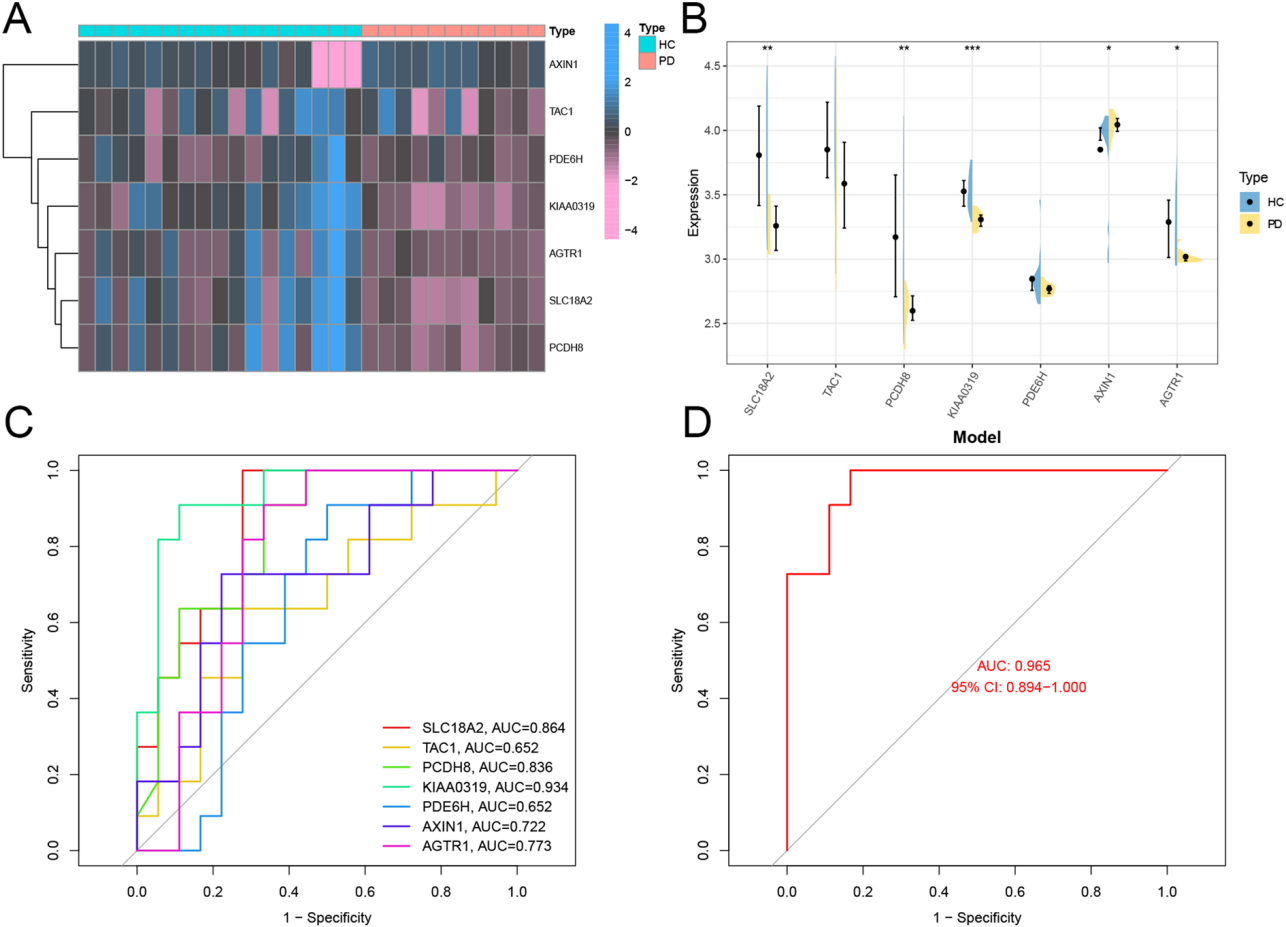
Validation of hub genes. The heatmap (**A**) and violin plot (**B**) of hub genes in HC and PD patients in the GSE20292 dataset. *P < 0.05, ** *P* < 0.01, *** *P* < 0.001. (**C**) Diagnostic value of 7 hub genes with ROC curves in GSE20292 dataset. (**D**) AUC area under the ROC curve.

To assess the clinical efficacy of our diagnostic model, we assessed it using SN datasets (GSE20292). Then, we developed a heatmap and a violin plot between HC and PD patients for the seven hub genes (Fig. 7A,B). Hub gene expression levels in the dataset were calculated using the ROC curves to identify the corresponding area under the curve (AUC). In Fig. 7C, AUCs for *SLC18A2*, *TAC1*, *PCDH8*, *KIAA0319*, *PDE6H*, *AXIN1*, and *AGTR1* in HC and PD patients were 0.864, 0.652, 0.863, 0.934, 0.65, 0.722, and 0.773, respectively. When the seven hub genes were combined into a single variable, their predictive value improved; the AUC for the model was 0.965 (Fig. 7D). Thus, the hub genes have a high predictive accuracy for diagnosis.

### Validation of expression level of the seven hub genes in peripheral blood samples.

**Figure 8:**
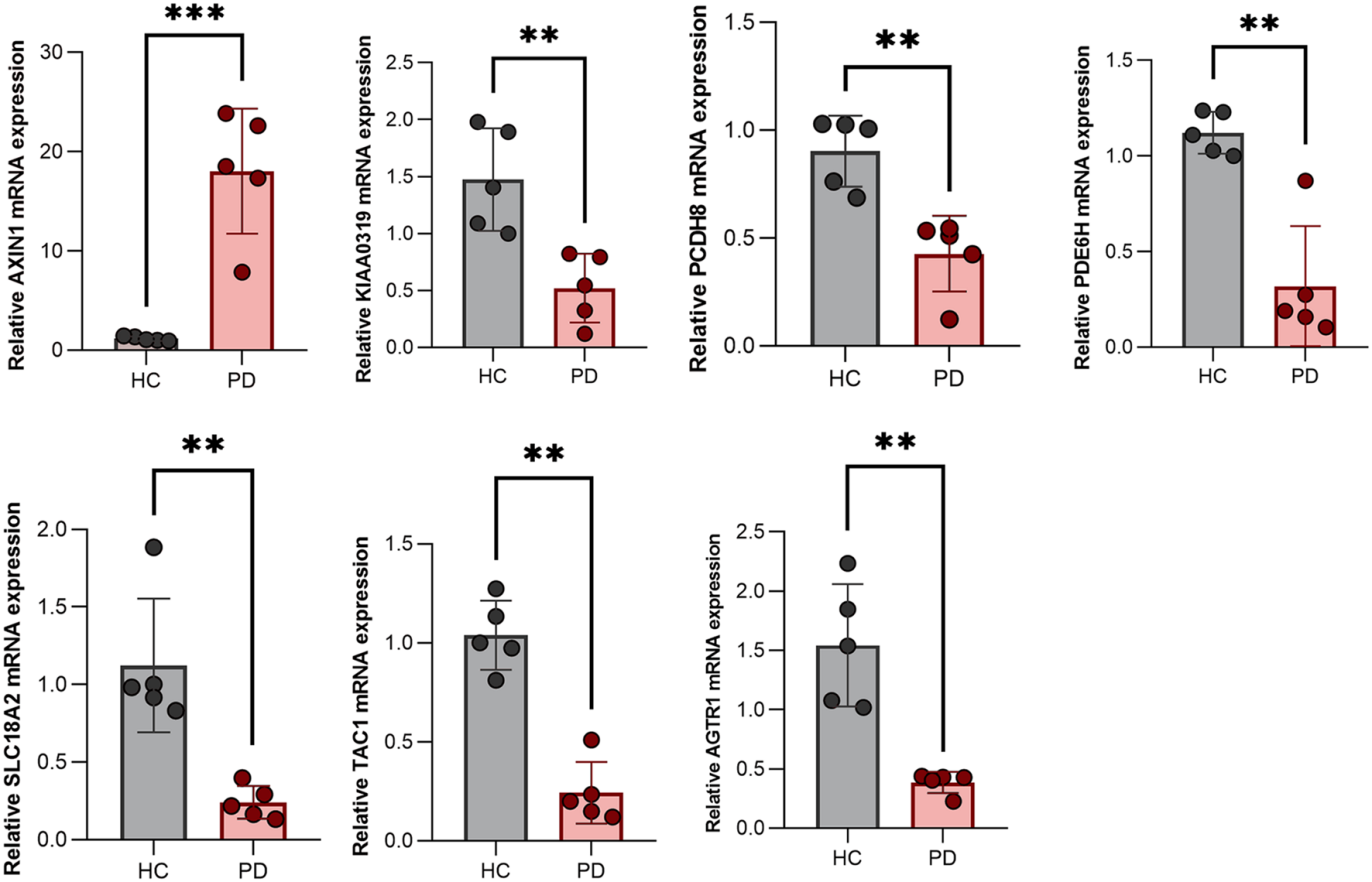
Expression level of SLC18A2, TAC1, PCDH8, KIAA0319, PDE6H, AXIN1, and AGTR1 in peripheral blood of HC and PD patients.

The RT-qPCR assay was performed to validate the efficacy of the prediction model. Relative expressions of *TAC1*, *PDE6H*, *KIAA0319*, *AGTR1*, *SLC18A2*, and *PCDH8* in HC were significantly higher than those in PD patients, while those of *AXIN1* were significantly lower in HC, relative to PD patients (Fig. 8). These results from peripheral blood samples are consistent with the GSE20292 dataset performed on SN, indicating that these genes hold promise as prospective therapeutic targets for PD patients.

## Discussion

The gold standard for diagnosing PD relies on the presence of SN pars compacta degeneration and Lewy pathology, as confirmed by post-mortem pathological examination^23,24^. Pathologically, PD is a slowly progressing neurological disease that begins years before a diagnosis^25^. Diagnostic examinations that allow for clear diagnosis during the initial phases of the disease, in particular, do not exist. Therefore, there is a need to develop suitable diagnostic approaches for early diagnosis. Through bioinformatics analyses, we aimed at identifying diagnostic genes that are related to the disease.

In previous studies, we acknowledge that some datasets were utilized in prior research by Zheng et al.^26^ and Kelly et al^27^. Zheng ’s study conducted a genome-wide meta-analysis of gene sets in PD, identifying 10 gene sets associated with the disease. Key findings include defects in mitochondrial electron transport, glucose utilization, and glucose sensing occurring early in disease pathogenesis. In Kelly’s study, DEGs were found to be associated with perturbed pathways, including mitochondrial dysfunction and oxidative stress. In our study, based on GSE7621, GSE20141, GSE20159, GSE20163, and GSE20164, RRA and batch correction were performed to identify the consensus DEGs, which were 80 in number. Among them, 71 genes were downregulated while nine genes were upregulated. Functional enrichment analysis revealed that the enriched GO terms were mainly in nerve conduction, DA metabolism, and DA biosynthesis. Pathologically, PD is associated with both neuronal dysfunction and inflammation of the central nervous system, consistent with nerve conduction disorders^28^. Dysregulated DA is more likely to play a crucial role in early onset of PD, thus, early identification of dysregulated DA should be a priority^29^. The enriched KEGG terms were also mainly in nerve conduction. Analysis of the PPI network revealed 39 hub genes, which were visualized by Cytoscape. The Lasso Cox regression model was used to assess the diagnostic genes with a high accuracy, which were *SLC18A2*, *TAC1*, *PCDH8*, *KIAA0319*, *PDE6H*, *AXIN1*, and *AGTR1*. CIBERSORT was used to assess immune cell infiltrations in PD. Plasma cells were found to be differentially expressed between HC and PD patients. Compared with previous studies, our study integrates multiple datasets and employs advanced methods such as RRA analysis and immune cell infiltration analysis, we believe that our work contributes to a more comprehensive understanding of the molecular mechanisms underlying PD.

*SLC18A2*, the vesicular monoamine transporter 2, is important in neurotransmitter transportation. It packages histamine into vesicles in preparation for neurotransmitter release from the presynaptic neuron^30^. The gene, which is important in the monoaminergic signaling pathway, has been extensively researched on. In the absence of this monoaminergic transporter, histamine immunoreactivity is significantly suppressed in neuronal cell bodies of the brain^31^. Reduced histamine metabolism in the central nervous system is a preventative measure against PD onset^32^. Further elucidation of the involved mechanisms will contribute to a better understanding of the disease. Substance P (SP) dysregulation is associated with the etiology of PD. Mice lacking endogenous SP (*TAC*1-/-) exhibited greater resistance to nigral dopaminergic neurodegeneration than wild-type controls. The neuroinflammatory and dopaminergic neurodegenerative effects of SP are mediated by microglial NOX2^33^, thus, they may shed new light on PD pathophysiology. Protocadherins, which contain *PCDH8*, are calcium-dependent adhesion molecules that have drawn interest for their potential functions in development of neural circuits and their potential effects on neurological illnesses. Physiologically, *PCDH8* is involved in development and maintenance of intrahippocampal circuits^34^. However, the association between *PCDH8* and PD has yet to be established. *KIAA0319* is associated with extracellular signaling pathways^35,36^. Extracellular signaling pathways and endocytosis are both necessary to control neurogenesis^37^. As a pivotal upstream gatekeeper, KIAA0319 plays crucial roles in neurogenesis by arresting cellular progression at the neural progenitor cell stage. Cell cycle progression is deregulated in PD, and key regulators of the G1/S transition checkpoint are significantly altered^38^. Moreover, the cell cycle is enriched in GO terms. Even though there is no conclusive proof that *KIAA0319* is directly associated with PD, our findings shed light on the subject. *PDE6H* is associated with changes in circadian rhythms that are involved in aging^39^, however, the association between PDE6H and PD has not been determined. *AXIN1* was overexpressed in hippocampus tissues and cells from MPTP-lesioned mice models of PD. *AXIN1* suppression in PD suppressed hippocampus neuron apoptosis. *AXIN1* downregulation suppresses DA neuron death via miR-128^40^. These outcomes suggest a therapeutic potential for *AXIN1* in treatment of PD. AGTR1 encodes the angiotensin II type 1 receptor (AT1R), whose expressions decreases in dopaminergic neurons of the SN as PD advances^41^. In contrast, AT1R upregulation induces the release of pro-inflammatory cytokines, leading to inflammation that culminates in dopaminergic cell death and dysfunction. Blocking AT1R with its antagonist can attenuate neurotoxin-induced degeneration of dopaminergic neurons in the SN^42,43^. These findings underscore the complex nature of AGTR1’s role in PD and highlight the need for further research.

The GO function analysis revealed that the DEGs were primarily enriched in nerve conduction, DA metabolism, and DA biosynthesis. Nerve conduction is divided into sensory nerve conduction and motor nerve conduction^44^. Regarding nerve conduction, Toth reported that individuals with PD exhibited slower motor conduction velocities, when compared to HC. Toth hypothesized that peripheral neuropathy in PD could be attributed to levodopa exposure and elevated levels of methylmalonic acid^45^. Thus, motor nerve transmission abnormalities maybe present in PD patients^46^. Pathologically, PD is characterized by degeneration of the nigrostriatal dopaminergic system. In 1988, Gotham et al. proposed the ‘dopamine overdose’ hypothesis^47^. The hypothesis, which proposes an impact on cognitive functions in PD, suggests that the medication doses required to restore DA functions in the most severely affected regions may be too high for less affected areas. According to this theory, the ventral striatum, which remains relatively intact, can become excessively stimulated when DA replacement therapy is administered. Subsequently, this overstimulation affects the limbic system, leading to impaired executive functions mediated by the limbic and orbitofrontal systems, such as learning and risk-taking. Empirical evidence from literature and clinical observations, particularly in relation to DA agonists, provides support for this theory and the emergence of impulse control disorders^48^. The enriched KEGG terms also included the PD and cycle of nerve conduction. The diagnostic gene, *SLC18A2,* is also associated with neurotransmitter transport, synaptic vesicle cycle, and dopaminergic synapses. In terms of neurotransmitter transport, mutations in *SLC18A2* will impact the transmission of monoamine neurotransmitters, leading to a phenotype that shares characteristics with all monoamine-related disorders^49^. In terms of the relationship between *SLC18A2* and DA, *SLC18A2* is a vesicular monoamine transporter that is essential in DA regulation. Suppressed *SLC18A2* activities might reduce DA release^50^.

Studies have reported on significantly changed B cell subpopulation structures in PD^51^. Given that plasma cells are derived from B cells, the abundance of plasma cells is associated with PD development. Plasma cells may affect PD pathogenesis by influencing the immune microenvironment. However, the relationship between plasma cells and PD has not been fully defined. Systematic studies should be performed to elucidate on possible mechanisms of plasma cells in PD.

Recognizing the practical challenges of obtaining brain tissue for routine diagnosis, we considered the more accessible and commonly used sample type, namely peripheral blood. Our intention was to validate the identified hub genes, derived from PD patients’ brain tissues, in peripheral blood. This approach is motivated by the fact that obtaining brain tissue for diagnosing suspected PD patients is impractical in routine clinical settings. Peripheral blood, being a commonly used and less invasive sample, is more feasible for routine diagnostic checks. We aimed to assess whether the gene expression patterns in blood align with those in brain tissues, with the goal of reducing diagnostic complexity and improving efficiency. However, our study has some limitations. First, even though we combined the datasets due to the lower number of samples in PD, studies with bigger sample sizes should be performed to confirm our findings. Second, we utilized RT-qPCR to validate the conclusions in blood samples of PD patients. Other experimental verification methods should be used to verify our results. Nowadays, larger-scale single cell RNA sequencing analysis and multi-omics with more clinical samples is needed to further elucidate the exact mechanism of the disease. We can make it realize in PD in the further research.

In conclusion, the developed diagnostic model provides new insights for early stage PD diagnosis. Plasma cells were found to be differentially expressed between HC and PD patients. Elucidation of the genetic and immunological mechanisms that underlie the initial signs of PD will unlock new therapeutic avenues. These insights will empower clinicians to effectively intervene with innovative or repurposed anti-inflammatory and immunomodulatory treatments, with the aim of slowing down the progression of this disease.

## Conclusion

The *SLC18A2*, *TAC1*, *PCDH8*, *KIAA0319*, *PDE6H*, *AXIN1*, and *AGTR1* are associated with PD pathology, and are potential diagnostic markers for PD. Besides, immune cell infiltrations might play an important role in PD. These findings hold promising implications for PD diagnosis and treatment.

### Supplementary Information

Supplementary Figure S1.

Supplementary Table S1.

Supplementary Table S2.

## Data Availability

All sequencing data generated in this study are deposited in the GEO database. (GSE7672, GSE20141, GSE20159, GSE20163, GSE20164, and GSE20292).

## Abbreviations

3D: 3 Dimension
AT1R: Angiotensin II type 1 receptor
AUC: Area under the curve
BP: Biological process
CC: Cellular component
DA: Dopamine
DEGs: Differentially expressed genes
FC: Fold change
GEO: Gene expression omnibus
GO: Gene ontology
HC: Healthy control
KEGG: Kyoto encyclopedia of genes and genomes
MF: Molecular function
PCA: Principal component analysis
PD: Parkinson's disease
PPI: Protein–protein interaction
ROC: Receiver operating characteristic
RRA: Robust rank aggregation
RT-qPCR: Reverse transcription-quantitative real-time polymerase chain reaction
SN: Substantia nigra
SP: Substance *P*
