# Supplementary figures and images for: Integrative analysis of gene expression profiles of substantia nigra identifies potential diagnosis biomarkers in Parkinson's disease
Source: Sci Rep. 2024 Jan 25;14:2167. doi: 10.1038/s41598-024-52276-0 (PMC10810830; doi:10.1038/s41598-024-52276-0)

**Supplementary Figure S1** Networks of the genes for each approach in cytoHubba plugin.


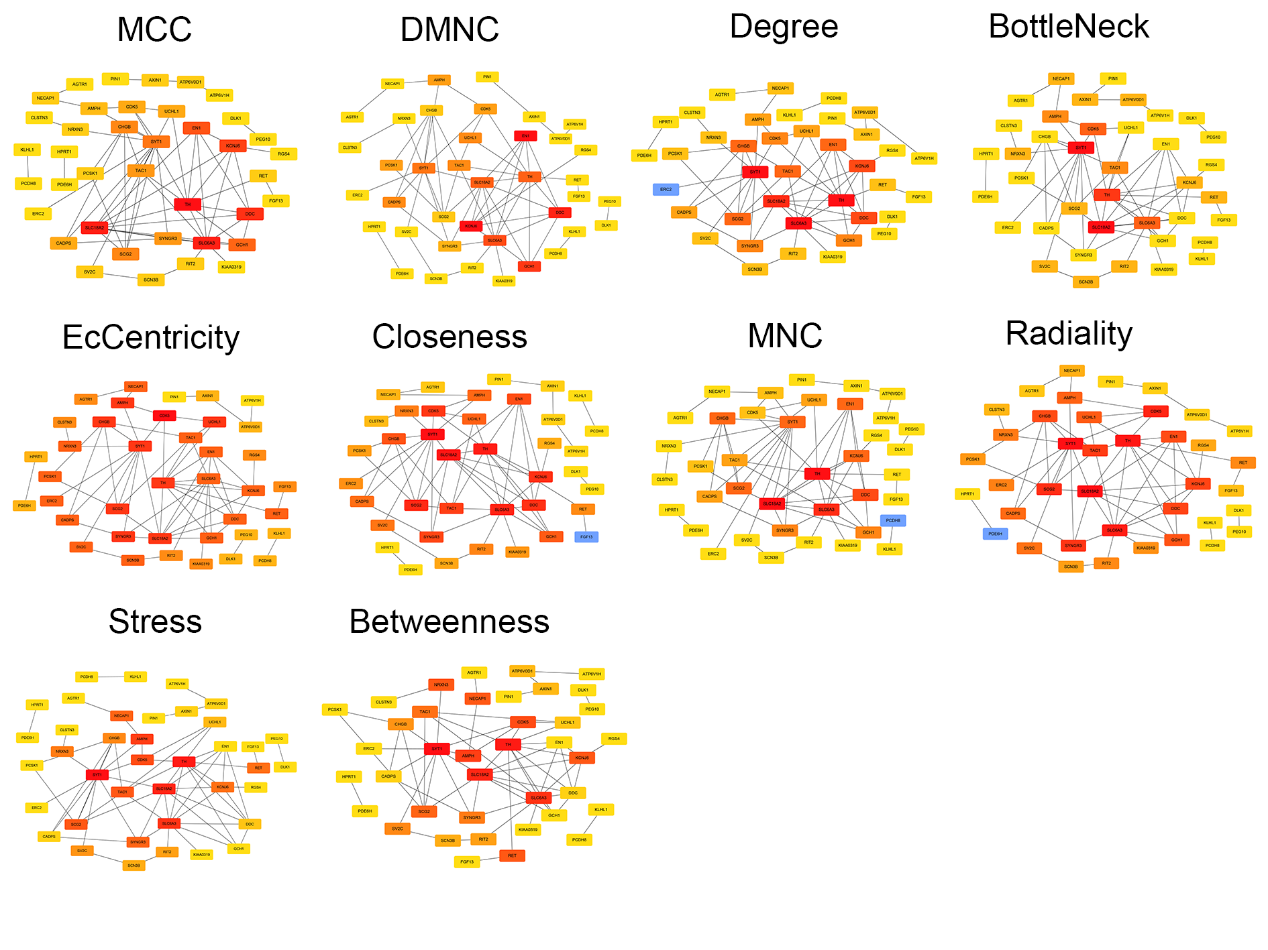

Supplement: Supplementary file 1 — Supplementary Figure S1. [file 41598_2024_52276_MOESM1_ESM.docx]
