# Supplementary material for: Integrative analysis of gene expression profiles of substantia nigra identifies potential diagnosis biomarkers in Parkinson's disease
Source: Sci Rep. 2024 Jan 25;14:2167. doi: 10.1038/s41598-024-52276-0 (PMC10810830; doi:10.1038/s41598-024-52276-0)
Supplement: Supplementary file 2 — Supplementary Table S1. [file 41598_2024_52276_MOESM2_ESM.docx]

**Supplementary Table S1** Clinical information for PD patients used in the RT-qPCR.

|  | Age | Sex | Diagnosis | Sample | Country |
| --- | --- | --- | --- | --- | --- |
| Patient 1 | 76 | male | PD | peripheral blood | China |
| Patient 2 | 67 | male | PD | peripheral blood | China |
| Patient 3 | 79 | female | PD | peripheral blood | China |
| Patient 4 | 66 | female | PD | peripheral blood | China |
| Patient 5 | 85 | female | PD | peripheral blood | China |
