# Supplementary material for: Integrative analysis of gene expression profiles of substantia nigra identifies potential diagnosis biomarkers in Parkinson's disease
Source: Sci Rep. 2024 Jan 25;14:2167. doi: 10.1038/s41598-024-52276-0 (PMC10810830; doi:10.1038/s41598-024-52276-0)
Supplement: Supplementary file 3 — Supplementary Table S2. [file 41598_2024_52276_MOESM3_ESM.docx]

**Supplementary Table S2** Genes ranked for each approach in cytoHubba.

Genes ranked by MCC method

| Rank | Name | Score |
| --- | --- | --- |
| 1 | TH | 151.0 |
| 2 | SLC6A3 | 150.0 |
| 2 | SLC18A2 | 150.0 |
| 4 | DDC | 144.0 |
| 5 | KCNJ6 | 121.0 |
| 6 | EN1 | 120.0 |
| 7 | GCH1 | 24.0 |
| 8 | SYT1 | 15.0 |
| 9 | CHGB | 8.0 |
| 9 | SCG2 | 8.0 |
| 11 | SYNGR3 | 6.0 |
| 11 | TAC1 | 6.0 |
| 13 | CDK5 | 4.0 |
| 13 | CADPS | 4.0 |
| 13 | UCHL1 | 4.0 |
| 16 | AMPH | 3.0 |
| 17 | ATP6V0D1 | 2.0 |
| 17 | AXIN1 | 2.0 |
| 17 | RIT2 | 2.0 |
| 17 | SCN3B | 2.0 |
| 17 | SV2C | 2.0 |
| 17 | RET | 2.0 |
| 17 | PCSK1 | 2.0 |
| 17 | NECAP1 | 2.0 |
| 17 | NRXN3 | 2.0 |
| 26 | RGS4 | 1.0 |
| 26 | KIAA0319 | 1.0 |
| 26 | KLHL1 | 1.0 |
| 26 | PCDH8 | 1.0 |
| 26 | ATP6V1H | 1.0 |
| 26 | PIN1 | 1.0 |
| 26 | DLK1 | 1.0 |
| 26 | PEG10 | 1.0 |
| 26 | ERC2 | 1.0 |
| 26 | FGF13 | 1.0 |
| 26 | HPRT1 | 1.0 |
| 26 | AGTR1 | 1.0 |
| 26 | PDE6H | 1.0 |
| 26 | CLSTN3 | 1.0 |

Genes ranked by DMNC method

| Rank | Name | Score |
| --- | --- | --- |
| 1 | EN1 | 0.6 |
| 1 | KCNJ6 | 0.6 |
| 3 | DDC | 0.6 |
| 4 | GCH1 | 0.6 |
| 5 | SLC6A3 | 0.4 |
| 6 | SLC18A2 | 0.4 |
| 6 | TH | 0.4 |
| 8 | SYT1 | 0.3 |
| 9 | CADPS | 0.3 |
| 9 | UCHL1 | 0.3 |
| 9 | TAC1 | 0.3 |
| 12 | CDK5 | 0.3 |
| 12 | PCSK1 | 0.3 |
| 12 | AMPH | 0.3 |
| 15 | SYNGR3 | 0.3 |
| 16 | CHGB | 0.3 |
| 16 | SCG2 | 0.3 |
| 18 | RGS4 | 0.0 |
| 18 | KIAA0319 | 0.0 |
| 18 | ATP6V0D1 | 0.0 |
| 18 | KLHL1 | 0.0 |
| 18 | AXIN1 | 0.0 |
| 18 | PCDH8 | 0.0 |
| 18 | ATP6V1H | 0.0 |
| 18 | PIN1 | 0.0 |
| 18 | RIT2 | 0.0 |
| 18 | DLK1 | 0.0 |
| 18 | SCN3B | 0.0 |
| 18 | PEG10 | 0.0 |
| 18 | SV2C | 0.0 |
| 18 | ERC2 | 0.0 |
| 18 | FGF13 | 0.0 |
| 18 | RET | 0.0 |
| 18 | HPRT1 | 0.0 |
| 18 | AGTR1 | 0.0 |
| 18 | PDE6H | 0.0 |
| 18 | NECAP1 | 0.0 |
| 18 | CLSTN3 | 0.0 |
| 18 | NRXN3 | 0.0 |

Genes ranked by Degree method

| Rank | Name | Score |
| --- | --- | --- |
| 1 | SLC6A3 | 10.0 |
| 1 | TH | 10.0 |
| 1 | SYT1 | 10.0 |
| 4 | SLC18A2 | 9.0 |
| 5 | DDC | 6.0 |
| 5 | KCNJ6 | 6.0 |
| 7 | EN1 | 5.0 |
| 7 | CHGB | 5.0 |
| 7 | SCG2 | 5.0 |
| 7 | TAC1 | 5.0 |
| 11 | CDK5 | 4.0 |
| 11 | GCH1 | 4.0 |
| 11 | SYNGR3 | 4.0 |
| 14 | CADPS | 3.0 |
| 14 | UCHL1 | 3.0 |
| 14 | AMPH | 3.0 |
| 17 | ATP6V0D1 | 2.0 |
| 17 | AXIN1 | 2.0 |
| 17 | RIT2 | 2.0 |
| 17 | SCN3B | 2.0 |
| 17 | SV2C | 2.0 |
| 17 | RET | 2.0 |
| 17 | PCSK1 | 2.0 |
| 17 | NECAP1 | 2.0 |
| 17 | NRXN3 | 2.0 |
| 26 | RGS4 | 1.0 |
| 26 | KIAA0319 | 1.0 |
| 26 | KLHL1 | 1.0 |
| 26 | PCDH8 | 1.0 |
| 26 | ATP6V1H | 1.0 |
| 26 | PIN1 | 1.0 |
| 26 | DLK1 | 1.0 |
| 26 | PEG10 | 1.0 |
| 26 | ERC2 | 1.0 |
| 26 | FGF13 | 1.0 |
| 26 | HPRT1 | 1.0 |
| 26 | AGTR1 | 1.0 |
| 26 | PDE6H | 1.0 |
| 26 | CLSTN3 | 1.0 |

Genes ranked by BottleNeck method

| Rank | Name | Score |
| --- | --- | --- |
| 1 | SLC18A2 | 17.0 |
| 1 | SYT1 | 17.0 |
| 3 | TH | 12.0 |
| 4 | SLC6A3 | 5.0 |
| 4 | CDK5 | 5.0 |
| 6 | TAC1 | 3.0 |
| 6 | AMPH | 3.0 |
| 8 | ATP6V0D1 | 2.0 |
| 8 | AXIN1 | 2.0 |
| 8 | KCNJ6 | 2.0 |
| 8 | RIT2 | 2.0 |
| 8 | SCN3B | 2.0 |
| 8 | SV2C | 2.0 |
| 8 | RET | 2.0 |
| 8 | SCG2 | 2.0 |
| 8 | NECAP1 | 2.0 |
| 8 | NRXN3 | 2.0 |
| 18 | DDC | 1.0 |
| 18 | RGS4 | 1.0 |
| 18 | KIAA0319 | 1.0 |
| 18 | EN1 | 1.0 |
| 18 | KLHL1 | 1.0 |
| 18 | PCDH8 | 1.0 |
| 18 | ATP6V1H | 1.0 |
| 18 | PIN1 | 1.0 |
| 18 | GCH1 | 1.0 |
| 18 | CADPS | 1.0 |
| 18 | DLK1 | 1.0 |
| 18 | SYNGR3 | 1.0 |
| 18 | PEG10 | 1.0 |
| 18 | CHGB | 1.0 |
| 18 | UCHL1 | 1.0 |
| 18 | ERC2 | 1.0 |
| 18 | FGF13 | 1.0 |
| 18 | PCSK1 | 1.0 |
| 18 | HPRT1 | 1.0 |
| 18 | AGTR1 | 1.0 |
| 18 | PDE6H | 1.0 |
| 18 | CLSTN3 | 1.0 |

Genes ranked by EcCentricity method

| Rank | Name | Score |
| --- | --- | --- |
| 1 | CDK5 | 0.2 |
| 2 | SLC18A2 | 0.2 |
| 2 | SYNGR3 | 0.2 |
| 2 | CHGB | 0.2 |
| 2 | UCHL1 | 0.2 |
| 2 | TH | 0.2 |
| 2 | SCG2 | 0.2 |
| 2 | AMPH | 0.2 |
| 2 | SYT1 | 0.2 |
| 10 | DDC | 0.1 |
| 10 | SLC6A3 | 0.1 |
| 10 | EN1 | 0.1 |
| 10 | KCNJ6 | 0.1 |
| 10 | GCH1 | 0.1 |
| 10 | CADPS | 0.1 |
| 10 | SCN3B | 0.1 |
| 10 | SV2C | 0.1 |
| 10 | ERC2 | 0.1 |
| 10 | RET | 0.1 |
| 10 | PCSK1 | 0.1 |
| 10 | TAC1 | 0.1 |
| 10 | NECAP1 | 0.1 |
| 10 | NRXN3 | 0.1 |
| 24 | RGS4 | 0.1 |
| 24 | KIAA0319 | 0.1 |
| 24 | RIT2 | 0.1 |
| 24 | FGF13 | 0.1 |
| 24 | AGTR1 | 0.1 |
| 24 | CLSTN3 | 0.1 |
| 30 | ATP6V0D1 | 0.1 |
| 30 | KLHL1 | 0.1 |
| 30 | AXIN1 | 0.1 |
| 30 | PCDH8 | 0.1 |
| 30 | DLK1 | 0.1 |
| 30 | PEG10 | 0.1 |
| 30 | HPRT1 | 0.1 |
| 30 | PDE6H | 0.1 |
| 38 | ATP6V1H | 0.0 |
| 38 | PIN1 | 0.0 |

Genes ranked by Closeness method

| Rank | Name | Score |
| --- | --- | --- |
| 1 | SYT1 | 17.9 |
| 2 | SLC18A2 | 17.6 |
| 3 | TH | 17.3 |
| 4 | SLC6A3 | 17.2 |
| 5 | SCG2 | 14.8 |
| 6 | DDC | 14.7 |
| 6 | KCNJ6 | 14.7 |
| 8 | CDK5 | 14.7 |
| 9 | TAC1 | 14.5 |
| 10 | SYNGR3 | 14.3 |
| 11 | EN1 | 14.2 |
| 12 | CHGB | 14.1 |
| 13 | GCH1 | 13.5 |
| 14 | UCHL1 | 12.9 |
| 15 | AMPH | 12.8 |
| 16 | CADPS | 12.6 |
| 17 | SV2C | 11.9 |
| 18 | NRXN3 | 11.6 |
| 19 | RIT2 | 11.5 |
| 20 | RET | 11.3 |
| 21 | ERC2 | 11.0 |
| 22 | KIAA0319 | 10.6 |
| 23 | PCSK1 | 10.5 |
| 24 | SCN3B | 10.2 |
| 25 | RGS4 | 9.7 |
| 26 | NECAP1 | 9.6 |
| 27 | CLSTN3 | 8.4 |
| 28 | FGF13 | 8.2 |
| 29 | AGTR1 | 7.3 |
| 30 | ATP6V0D1 | 2.5 |
| 30 | AXIN1 | 2.5 |
| 32 | ATP6V1H | 1.8 |
| 32 | PIN1 | 1.8 |
| 34 | KLHL1 | 1.0 |
| 34 | PCDH8 | 1.0 |
| 34 | DLK1 | 1.0 |
| 34 | PEG10 | 1.0 |
| 34 | HPRT1 | 1.0 |
| 34 | PDE6H | 1.0 |

Genes ranked by MNC method

| Rank | Name | Score |
| --- | --- | --- |
| 1 | SLC18A2 | 9.0 |
| 1 | TH | 9.0 |
| 3 | SLC6A3 | 8.0 |
| 4 | DDC | 6.0 |
| 5 | EN1 | 5.0 |
| 5 | KCNJ6 | 5.0 |
| 5 | CHGB | 5.0 |
| 5 | SCG2 | 5.0 |
| 5 | SYT1 | 5.0 |
| 10 | GCH1 | 4.0 |
| 10 | SYNGR3 | 4.0 |
| 12 | CADPS | 3.0 |
| 12 | UCHL1 | 3.0 |
| 12 | TAC1 | 3.0 |
| 15 | CDK5 | 2.0 |
| 15 | PCSK1 | 2.0 |
| 15 | AMPH | 2.0 |
| 18 | RGS4 | 1.0 |
| 18 | KIAA0319 | 1.0 |
| 18 | ATP6V0D1 | 1.0 |
| 18 | KLHL1 | 1.0 |
| 18 | AXIN1 | 1.0 |
| 18 | PCDH8 | 1.0 |
| 18 | ATP6V1H | 1.0 |
| 18 | PIN1 | 1.0 |
| 18 | RIT2 | 1.0 |
| 18 | DLK1 | 1.0 |
| 18 | SCN3B | 1.0 |
| 18 | PEG10 | 1.0 |
| 18 | SV2C | 1.0 |
| 18 | ERC2 | 1.0 |
| 18 | FGF13 | 1.0 |
| 18 | RET | 1.0 |
| 18 | HPRT1 | 1.0 |
| 18 | AGTR1 | 1.0 |
| 18 | PDE6H | 1.0 |
| 18 | NECAP1 | 1.0 |
| 18 | CLSTN3 | 1.0 |
| 18 | NRXN3 | 1.0 |

Genes ranked by Radiality method

| Rank | Name | Score |
| --- | --- | --- |
| 1 | SLC18A2 | 4.0 |
| 1 | SYT1 | 4.0 |
| 3 | TH | 3.9 |
| 4 | SLC6A3 | 3.8 |
| 5 | CDK5 | 3.8 |
| 6 | SCG2 | 3.7 |
| 7 | SYNGR3 | 3.7 |
| 8 | TAC1 | 3.7 |
| 9 | DDC | 3.6 |
| 9 | KCNJ6 | 3.6 |
| 11 | EN1 | 3.6 |
| 11 | CHGB | 3.6 |
| 13 | GCH1 | 3.6 |
| 14 | UCHL1 | 3.5 |
| 15 | AMPH | 3.5 |
| 16 | CADPS | 3.5 |
| 17 | SV2C | 3.4 |
| 18 | NRXN3 | 3.3 |
| 19 | ERC2 | 3.3 |
| 20 | RIT2 | 3.2 |
| 20 | RET | 3.2 |
| 22 | KIAA0319 | 3.1 |
| 23 | PCSK1 | 3.1 |
| 24 | SCN3B | 3.1 |
| 25 | RGS4 | 2.9 |
| 26 | NECAP1 | 2.8 |
| 27 | CLSTN3 | 2.6 |
| 28 | FGF13 | 2.5 |
| 29 | AGTR1 | 2.1 |
| 30 | ATP6V0D1 | 0.4 |
| 30 | AXIN1 | 0.4 |
| 32 | ATP6V1H | 0.3 |
| 32 | PIN1 | 0.3 |
| 34 | KLHL1 | 0.2 |
| 34 | PCDH8 | 0.2 |
| 34 | DLK1 | 0.2 |
| 34 | PEG10 | 0.2 |
| 34 | HPRT1 | 0.2 |
| 34 | PDE6H | 0.2 |

Genes ranked by Stress method

| Rank | Name | Score |
| --- | --- | --- |
| 1 | SYT1 | 480.0 |
| 2 | TH | 282.0 |
| 3 | SLC18A2 | 280.0 |
| 4 | SLC6A3 | 254.0 |
| 5 | AMPH | 168.0 |
| 6 | CDK5 | 136.0 |
| 7 | SCG2 | 94.0 |
| 7 | TAC1 | 94.0 |
| 9 | SYNGR3 | 86.0 |
| 9 | NECAP1 | 86.0 |
| 11 | KCNJ6 | 80.0 |
| 11 | RET | 80.0 |
| 13 | NRXN3 | 72.0 |
| 14 | CHGB | 70.0 |
| 15 | SV2C | 46.0 |
| 16 | RIT2 | 34.0 |
| 17 | SCN3B | 20.0 |
| 18 | UCHL1 | 8.0 |
| 19 | DDC | 6.0 |
| 20 | ATP6V0D1 | 4.0 |
| 20 | AXIN1 | 4.0 |
| 20 | CADPS | 4.0 |
| 23 | RGS4 | 0.0 |
| 23 | KIAA0319 | 0.0 |
| 23 | EN1 | 0.0 |
| 23 | KLHL1 | 0.0 |
| 23 | PCDH8 | 0.0 |
| 23 | ATP6V1H | 0.0 |
| 23 | PIN1 | 0.0 |
| 23 | GCH1 | 0.0 |
| 23 | DLK1 | 0.0 |
| 23 | PEG10 | 0.0 |
| 23 | ERC2 | 0.0 |
| 23 | FGF13 | 0.0 |
| 23 | PCSK1 | 0.0 |
| 23 | HPRT1 | 0.0 |
| 23 | AGTR1 | 0.0 |
| 23 | PDE6H | 0.0 |
| 23 | CLSTN3 | 0.0 |

Genes ranked by Betweenness method

| Rank | Name | Score |
| --- | --- | --- |
| 1 | SYT1 | 308.6 |
| 2 | TH | 170.3 |
| 3 | SLC18A2 | 153.1 |
| 4 | SLC6A3 | 144.4 |
| 5 | AMPH | 104.0 |
| 6 | CDK5 | 75.9 |
| 7 | KCNJ6 | 54.0 |
| 7 | RET | 54.0 |
| 7 | NECAP1 | 54.0 |
| 7 | NRXN3 | 54.0 |
| 11 | SCG2 | 43.7 |
| 12 | TAC1 | 43.4 |
| 13 | SYNGR3 | 33.6 |
| 14 | SV2C | 30.8 |
| 15 | CHGB | 30.7 |
| 16 | RIT2 | 26.7 |
| 17 | SCN3B | 7.5 |
| 18 | ATP6V0D1 | 4.0 |
| 18 | AXIN1 | 4.0 |
| 20 | UCHL1 | 2.5 |
| 21 | DDC | 1.5 |
| 21 | CADPS | 1.5 |
| 23 | RGS4 | 0.0 |
| 23 | KIAA0319 | 0.0 |
| 23 | EN1 | 0.0 |
| 23 | KLHL1 | 0.0 |
| 23 | PCDH8 | 0.0 |
| 23 | ATP6V1H | 0.0 |
| 23 | PIN1 | 0.0 |
| 23 | GCH1 | 0.0 |
| 23 | DLK1 | 0.0 |
| 23 | PEG10 | 0.0 |
| 23 | ERC2 | 0.0 |
| 23 | FGF13 | 0.0 |
| 23 | PCSK1 | 0.0 |
| 23 | HPRT1 | 0.0 |
| 23 | AGTR1 | 0.0 |
| 23 | PDE6H | 0.0 |
| 23 | CLSTN3 | 0.0 |
